# Prospective Audit of Blood Donor Selection Process in a Tertiary Care Hospital of a Developing Country

**DOI:** 10.4274/tjh.2015.0094

**Published:** 2016-05-16

**Authors:** Naila Raza

**Affiliations:** 1 Liaquat National Hospital & Medical College, Department of Hematology, Karachi, Pakistan

**Keywords:** Medical audit, Transfusion Medicine, Donor selection

## Abstract

**Objective:**

The aim of this study was to emphasize the significance of internal audits of the blood donor selection process and documentation in a resource-limited country by assessing compliance with the established protocols, and to identify weak areas in the process.

**Materials and Methods:**

This audit reviewed the donor selection process at the blood bank of Liaquat National Hospital & Medical College, Karachi, over a 6-month period. Seven variables selected as performance indicators were graded as very good (%90-100%), good (80%-89%), satisfactory (70%-79%), or unacceptable (<70%). Blood bank staff was asked for feedback and suggestions.

**Results:**

Documentation of donor demographics was not within the acceptable range (documentation rates of 65.14%), donor status records were satisfactory (77.64%), and donor physical exam records were graded as good (86.34%). Five performance indicators were graded as very good (90%-100%).

**Conclusion:**

The audit proved productive in identifying major causes of irregularities in documentation and in making valuable suggestions for their rectification.

## Abstract (tr)

**Amaç:**

Bu çalışmanın amacı kaynakları kısıtlı bir ülkede kan donörü seçimi sürecinin hastane içinde denetlenmesinin ve belgelenmesinin önemini vurgulamak ve sürecin zayıf noktalarının tespitini yapmaktır.

**Gereç ve Yöntemler:**

Bu denetleme ile 6 ay boyunca Liaquat National Hospital & Medical College, Karachi Kan Bankası’nda donör seçim süreci gözden geçirdi. Verimliliğini belirlemek için kullanılan 6 değişken şu şekilde derecelendirildi, çok iyi (%90-100), iyi (%80-89), yeterli (%70-79) ve kabul edilemez (<%70). Kan bankası çalışanlarına geri bildirimleri ve önerileri soruldu.

**Bulgular:**

Donör bilgilerinin belgelenmesi kabul edilebilir düzeyde değildi (%65,14), donörlerin durumunun kayıtları yeterliydi (%77,64), donörlerin fizik muayene kayıtları iyi olarak derecelendirildi (%86,34). Beş verimlilik belirteci çok iyi olarak derecelendirildi (%90-100).

**Sonuç:**

Denetleme belgelendirme konusundaki düzensizliğin ana sebeplerini belirlemede ve bunların düzeltilmesinde değerli önerilerde bulunma konusunda verimli oldu.

## INTRODUCTION

Documentation and record-keeping play integral roles in transfusion medicine from every step of the vein-to-vein chain of blood donation to the dispatch of blood components. Regular medical audits are a part of quality assurance programs in transfusion medicine and a means of continuous assessment and improvement of existing systems. For conducting audits of clinical laboratories, a written set of questions in the form of a checklist is used, evaluation of which indicates whether the laboratory is performing its procedures according to its documented policies and standard operating procedures and on time. Historically, audits done in blood banks were focused on clinical uses of blood components to ensure appropriate use, minimize wastage, and reduce the risk of transfusion-transmissible diseases. Developing countries like Pakistan depend heavily on non-remunerated blood donors as only 10% of blood donations are collected from voluntary donors [1]. Donor deferrals based on pre-donation assessment and workup acts as a deterrent for future donations, especially among first-time donors [2]. The World Health Organization (WHO) calls for a quality system to be put in place for blood donor selection criteria, staff training, and documentation [3]. A donor questionnaire is the key tool in donor selection for assessing donor health and safety and in reducing the risk of transmission of infections. Timely counseling with regular reminders can help in re-recruiting short-term temporarily deferred donors back into the donor pool. Prior to these efforts, we have to ensure that donor screening records are properly maintained. There are no published data on internal audits done on donor screening processes in Pakistan.

The objective of our study is to assess compliance with the established protocols for blood donor selection processes and documentation, to identify weak areas in these processes, and to recommend improvements in the system based on feedback obtained from blood bank staff.

## MATERIALS AND METHODS

**Figure 1:**
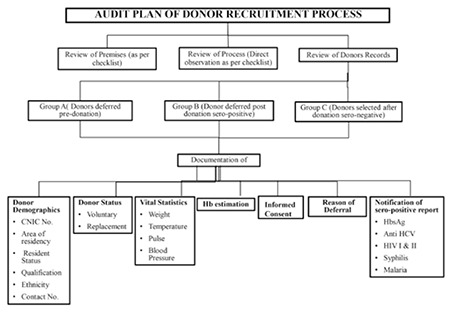
Audit plan.

As a part of quality system improvement we planned a prospective 6-month internal audit of the donor recruitment process and documentation at the blood bank of a tertiary care hospital in Karachi, Pakistan, from January to June 2014. An audit plan was devised and checklists were prepared with the help of a toolkit developed by the Directorate General of Health Services, Dhaka WHO, July 2008 [4]. The audit involved the review of premises and the donor selection process as per checklists and scrutiny of donor records for documentation. The audit plan and checklists are shown in Figure 1 and Table 1.

Donor records were grouped into group A (donors deferred before donation), group B (donors rejected after donation: seropositive cases), and group C (donors selected for donation: seronegative cases). We selected documentation of 7 parameters as performance indicators: donor demographics, donor status, general physical exam, hemoglobin estimation, informed consent, reason of deferral, and notification of seropositive cases. For each group, performance was graded as very good, good, satisfactory, or unacceptable by maintaining a high level of scoring documentation rates of 90%-100%, 80%-89%, 70%-79%, and <70%, respectively. Based on the results, feedback was obtained from the blood bank staff responsible for conducting interviews of donors to determine common causes of nonconformance. A list of recommendations for appropriate changes in the current system was designed and submitted to the head of the blood transfusion services of the institute at the end of this exercise.

The study was conducted after obtaining approval from the institute’s ethics review committee.

Data analysis was performed using descriptive statistics. Numerical data are shown as percentages.

## RESULTS

In this study, inspection of the donor area was found to be satisfactory as almost all the prerequisites mentioned in the checklist were met. Equipment and materials were being properly maintained as the department is ISO-9001:2000 certified. Exceptions were absence of privacy for asking questions related to sexual behavior and lack of written material for donor self-deferral. Staff members were qualified and trained. Review of donor records from January 2014 to June 2014 showed that out of 10,041 prospective blood donors, 1027 donors belonged to group A, 496 to group B, and 8518 to group C. Donor demographic records were inadequately maintained (documentation rate: 65.14%) as donor identification card numbers and area of residency were not always documented in all 3 groups. This was followed by donor status (documentation rate: 77.64%) and vital statistics (documentation rate: 86.34%) in that order. See Supplement 1 for review of Total Donor Screening Forms and Comparison of Documentation rate among 3 groups of Donor Selection Forms.

Among deferred donors, the reason of deferral was mentioned in all cases but the donor notification rate was 89.51%. The main reason cited for not documenting the identification card number was not asking for it due to its nonavailability at the time of donation, and the area of residency was missed due to ignorance about the exact zonal divisions of the city. Irregularities in documentation of donor’s vital statistics were mainly due to bypassing the set standard operating procedures.

## DISCUSSION

This audit gave insight into the existing practices of the donor selection process at our institute in particular and in developing countries in general. Random checks were conducted to evaluate the premises and processes using checklists and direct observations. Although the overall performance and documentations were good, some important issues were highlighted. Lack of privacy for conducting donor interviews was a concern identified in our study and mentioned by Kumar et al. in a similar study from India [5]. Absence of proper infrastructure and space limitations are common problems faced by health centers in developing countries.

Review of donor records showed some nonconformance in donor demographics including irregularities in donor identification numbers and area of residency in all 3 groups. The purpose of the former is donor traceability and that of the latter is use for epidemiological data. Use of mobile numbers for contacting donors has become the norm as it is much easier and a quick method for donor notification that can safely replace identification numbers. However, its documentation rate needs to be 100%, especially in deferred donors with seropositive status (group B); in our case, this rate was 94.50%. Area of residency has also lost credibility as people lack awareness of exact zonal locations due to formation of new localities and the constant expansion of the city.

Our study showed a shortcoming as per documentation rate for donor status (77%). Omission of data regarding donor status can be overcome by using different-colored forms for voluntary donors or by keeping separate registers. In this way, the focus can be directed towards voluntary donors with regular reminders for donations, thus facilitating the donor recruitment program. Documentation rate of vital signs collectively was good (86%). Documentation of the remaining 4 indicators was satisfactory.

Feedback from blood bank staff was obtained to determine the most likely causes for omitted data. Failure to document identification number and area of residency was due to a silent understanding among staff about their triviality; hence, this information was not being collected from donors. Donor status was not noted mostly due to inattention and the incongruous location of the question window in the proforma according to the staff. Documentation rate of vital statistics was selectively poor in group A (documentation rate: 59.59%). This was attributed to bypassing of normal standard operating procedures of conducting a physical exam first, followed by hemoglobin estimation, by some new staff members due to ignorance of the protocol. Notifying donors about the potential presence of transfusion-transmissible disease is a major responsibility of blood banks. In our study, the donor notification documentation rate was good (94.5%). Failure to inform donors were due to no response when called, wrong mobile numbers, and failure to document the mobile number, in that order. Thus, instead of identity card number, at least two contact phone numbers should be noted to ensure a 100% donor notification record.

## CONCLUSION AND RECOMMENDATIONS

The donor selection process is a vital link in the chain of blood collection, screening, and transfusion. A detailed audit of this program showed certain gaps in the documentation process. The following recommendations are made to minimize chances of lacunae and further improve the system:

• Privacy must be provided for conducting donor interviews.

• Donor identity card number and area of residency may be removed from the donor pro forma and replaced by two valid contact phone numbers.

• Separate registers or color-coded forms can be used for voluntary blood donors.

• Mini-audits of selected areas must be done apart from the annual external audits to improve quality.

• Refresher courses for blood bank staff should be conducted regularly.

• Introduction of electronic record-keeping in blood banks is vital for easy data retrieval.

## Ethics

Ethics Committee Approval: Ethical Review Committee, Liaquat National Hospital, Institute for Postgraduate Medical Studies and Health Sciences, Karachi, Pakistan (App No. 0181-2014 LNH-ERC). Informed Consent: It was taken.

## Figures and Tables

**Comparison:**
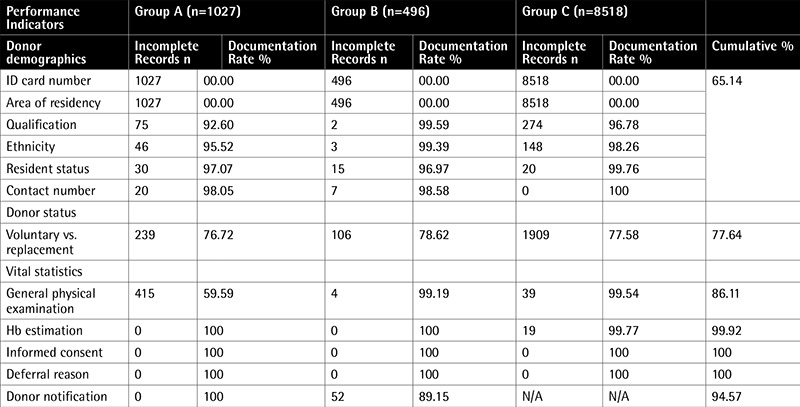
Comparison of documentation rate among 3 groups of donor selection forms.

**Supplement 1:**
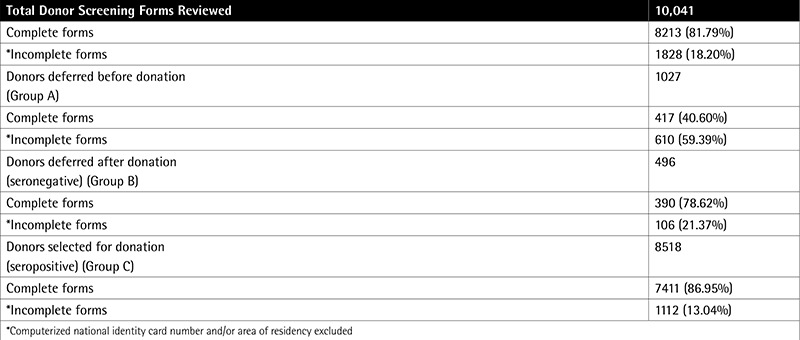
Comparison of documentation rate among 3 groups of donor selection forms and total donor screening forms reviewed. Audit of donor selection forms (January-June 2014).

**Table 1:**
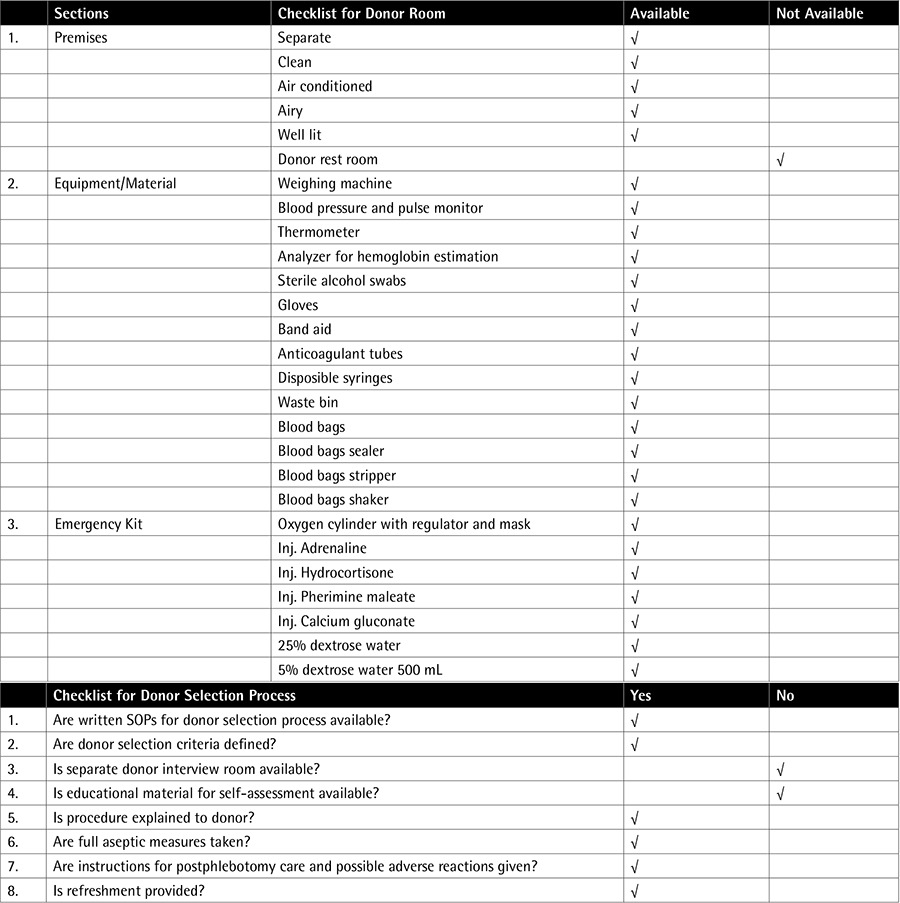
Checklists for donor selection.
